# Old enough to be a model? On the role of maturity in stem cell-based models for neuropsychiatric disorders

**DOI:** 10.1016/j.nsa.2026.106982

**Published:** 2026-01-22

**Authors:** Bingqing He, Erik Smedler

**Affiliations:** a Department of Psychiatry and Neurochemistry, Institute of Neuroscience and Physiology, Sahlgrenska Academy at University of Gothenburg, Gothenburg, Sweden; b The Wallenberg Centre for Molecular and Translational Medicine, Gothenburg, Sweden; c Department of Clinical Chemistry, Sahlgrenska University Hospital, Gothenburg, Sweden

## Abstract

Mental disorders profoundly influence cognition, emotion, and self-perception, and collectively represent a major cause of global disability. Their onset spans distinct developmental periods, from early childhood in neurodevelopmental conditions such as autism spectrum disorder, through adolescence in eating and obsessive-compulsive disorders, to early adulthood in bipolar disorder and schizophrenia. Twin and family studies have established that these disorders are substantially heritable, and large-scale genomic analyses have identified numerous common and rare risk variants. Yet, the biological mechanisms through which genetic and environmental factors converge to shape disease trajectories remain elusive. Patient-derived induced pluripotent stem cells (iPSCs) have emerged as a promising tool for investigating disease-relevant mechanisms in human neurons and neural circuits. However, most iPSC-derived neural cells and organoids resemble embryonic/fetal-stage brain tissue in both molecular and functional characteristics, raising questions about their relevance for disorders that manifest later in life. In this narrative review, we discuss how developmental timing, both in disease onset and in cellular models, shapes the interpretation of iPSC-based findings. We outline how differences in neuronal maturity may constrain or enable mechanistic insight, summarize emerging methods for accelerating or extending neuronal aging *in vitro*, and consider how leveraging developmental immaturity might illuminate early pathogenic processes underlying mental disorders.

## 1 Introduction

Patient-derived induced pluripotent stem cell (iPSC) models have become a central experimental platform for studying the biological mechanisms underlying neuropsychiatric disorders. These systems offer unprecedented access to human neurons carrying patient-specific genetic backgrounds, but they also present a fundamental challenge. Most iPSC-derived neural cells and organoids resemble embryonic or mid-fetal stages of human brain development. This raises a critical question for the field regarding whether current stem cell-based models are developmentally aligned with the biological processes they are intended to capture.

In this narrative review, we examine how developmental timing shapes both disease vulnerability and experimental model interpretation. We integrate epidemiological data on age of onset, genetic and transcriptomic studies of neurodevelopmental risk, and current knowledge of neuronal maturation in two- and three-dimensional iPSC-derived systems. We further discuss emerging strategies to accelerate, extend, or artificially modulate neuronal maturation *in vitro*, and consider how developmental immaturity may, in some contexts, represent a conceptual advantage rather than a limitation for modeling psychiatric disease mechanisms.

## 2 Literature search strategy

This narrative review is based on a targeted and selective literature search and does not aim to provide a comprehensive or systematic survey of all published work. Relevant studies were identified primarily through searches of PubMed using keywords related to iPSCs, neuronal maturation, brain organoids, neurodevelopment, and neuropsychiatric disorders, complemented by manual screening of reference lists and the authors’ expertise. The goal was to synthesize key conceptual and methodological insights rather than to exhaustively catalogue the literature.

## 3 Age of onset as a window into disease biology

**Fig. 1:**
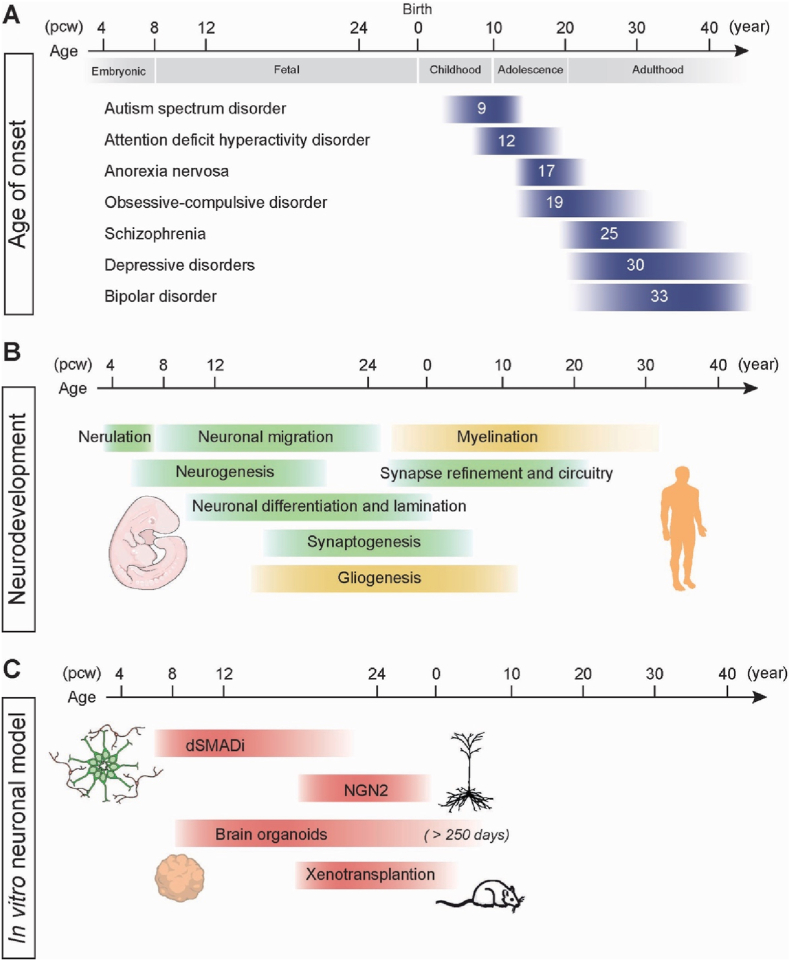
Temporal comparison of age of onset for mental disorders with neurodevelopmental processes and hPSC derived neuronal models. (A) Median age of onset for a subset of mental disorders. Data from (Solmi et al., 2022). (B) Timing of key developmental processes in the human brain. Data from (Zhou et al., 2024) (C) Corresponding age of different cell models.

Mental disorders show different developmental onsets, implying that the timing of vulnerability is biologically meaningful. A global meta-analysis of 192 epidemiological studies revealed median ages of onset ranging from 5 years for neurodevelopmental disorders to the early-late 20s for psychotic and mood disorders (Solmi et al., 2022). See Fig. 1A for examples of the age of onset as measured by median age from (Solmi et al., 2022). However, disease processes may start years before diagnosis, as exemplified in schizophrenia where soft neurological signs are sometimes seen during childhood and first psychosis is preceded by months to years of diffuse prodromal symptoms (Ferruccio et al., 2021; White et al., 2006). Another caveat is the potential delay of patients seeking psychiatric evaluation as well as doctor's delay in getting the right diagnosis. This is an issue especially in bipolar disorder, where evidence-based treatment with mood-stabilizers might be delayed up to ten years from onset of symptoms (Scott et al., 2022).

Another important caveat is that epidemiological estimates of age at onset should be interpreted with caution, particularly for neurodevelopmental disorders. Early manifestations are often subtle, nonspecific, or difficult to detect, and very young children have limited ability to articulate internal experiences. Consequently, clinical recognition and diagnosis may occur long after the underlying biological processes have begun, implying that the true onset of disease may precede the ages reported in population-based studies.

This diverse temporal staging of age of onset for mental disorders aligns with major neurodevelopmental milestones, for example synaptic pruning, myelination, and maturation of prefrontal–limbic circuits, that extend well into young adulthood (Marin, 2016; Paus et al., 2008) (see Fig. 1B). Human neural development is extensive compared to other mammals, with nine months long gestation, and postnatal development extending beyond 20 years of age (Molnar et al., 2019). This results in a highly complex organ, with a larger neocortical area than other mammals and more developed sulci and gyri. Multiple populations of progenitor cells in an extra outer subventricular zone and prolonged neurogenesis form the cellular basis of this complexity (Braun et al., 2023; Velmeshev et al., 2023; Wang et al., 2025; Zeng et al., 2023).

## 4 Genetic basis of mental disorders

Genetic evidence further supports the existence of temporally distinct etiological windows in mental disorders. The high heritability of conditions such as autism spectrum disorder (ASD), attention-deficit/hyperactivity disorder (ADHD), schizophrenia, and bipolar disorder has been reflected in the identification of extensive sets of risk variants through genome-wide association studies (GWAS) (Agrawal et al., 2025). Notably, the vast majority of these variants are located in non-coding regions of the genome, suggesting that they exert their effects primarily through the regulation of gene expression rather than alterations in protein sequence (Xiao et al., 2017). Integrating transcriptomic and epigenomic data from multiple human brain regions spanning prenatal to adult stages, the PsychENCODE project mapped how gene-expression programs change across development (Li et al., 2018). A striking transcriptional transition occurs during late fetal development, characterized by a transient reduction in regional heterogeneity and a coordinated shift toward mature neuronal gene-expression programs, alongside asynchronous changes in myelination-related processes. Co-expression module analyses revealed that genetic risk variants for neuropsychiatric disorders, including ASD and schizophrenia, converge within a limited number of developmentally dynamic modules that show their greatest rate of change during mid-to late fetal development (approximately 12–35 postconceptional weeks). These modules are highly enriched in cortical excitatory projection neurons, particularly deep-layer populations generated during mid-fetal corticogenesis, and include genes critical for neuronal differentiation, migration, and synapse formation, such as *MEF2C*, *SATB2*, and *TCF4*. Complementary epigenomic analyses demonstrated coordinated changes in enhancer-associated DNA methylation and histone modifications, consistent with dynamic chromatin remodeling during neural maturation. Collectively, these findings indicate that many psychiatric risk genes exert their effects during discrete prenatal windows of human brain development, with particular vulnerability during late fetal stages extending into early infancy.

Complementary studies integrating GWAS with single-cell transcriptomic data have further refined this view by mapping genetic risk to discrete neuronal populations (Yao et al., 2025). Across disorders, both excitatory and inhibitory neurons emerge as principal mediators of genetic risk (Bryois et al., 2020). In schizophrenia, common-variant enrichment is observed preferentially in pyramidal neurons, medium spiny neurons, and interneurons, rather than progenitor or glial populations (Skene et al., 2018). Together, these findings underscore that modelling neuropsychiatric disorders *in vitro* requires attention not only to developmental timing but also to the generation of specific disease-relevant neuronal subtypes. Because current iPSC-derived dorsal forebrain models most closely approximate early-to mid-fetal stages of human cortical development, they are well suited for studying early etiological mechanisms driven by prenatal genetic risk but may be limited in their ability to capture later aspects of neuronal maturation and circuit integration relevant to disease expression.

## 5 Developmental identity of iPSC-derived neural models

Because experimental access to living human brain tissue is extremely limited, most neurobiological research has relied on animal models to investigate gene-phenotype relationships. However, mental disorders are inherently human, and animal models can only approximate their biological complexity. The advent of human pluripotent stem cell technologies, particularly iPSCs, has transformed this landscape by enabling the generation and study of developing human neural tissue under controlled laboratory conditions (Yamanaka, 2012).

### 5.1 Two-dimensional cultures

**Fig. 2:**
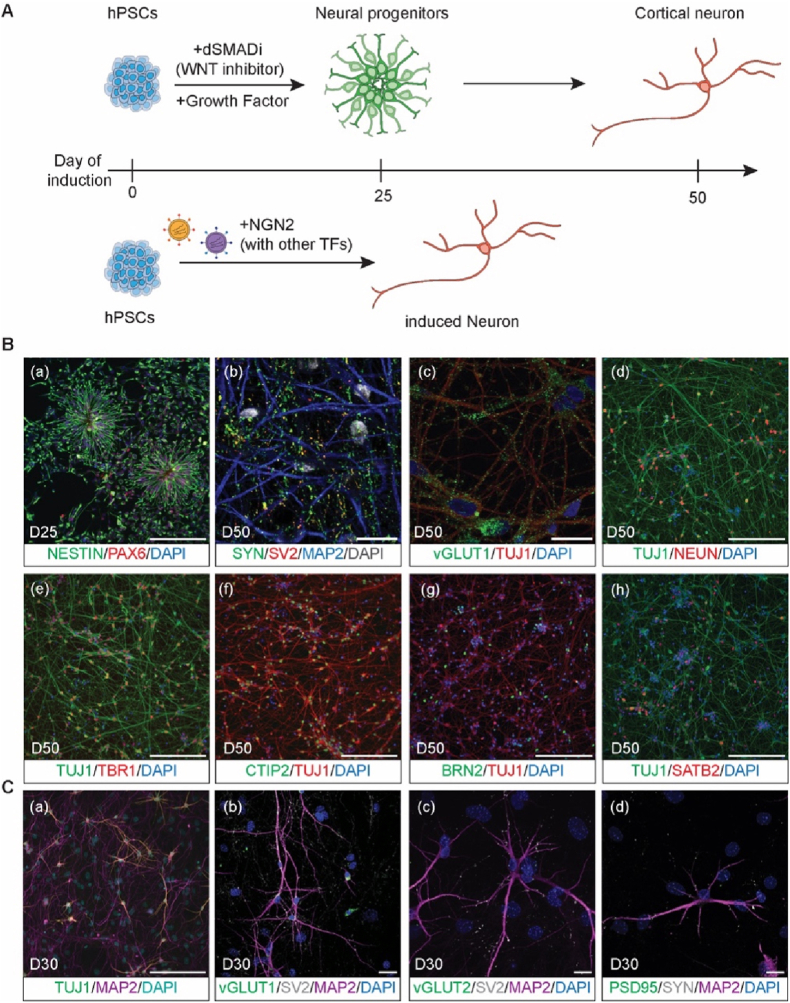
hPSC derived two-dimensional neuronal differentiation. (A) Schemes of cortical neuronal differentiation via dual SMAD-inhibition (dSMADi) method (upper) and NGN2-induced method (lower). (B) Representative immunofluorescence images for differentiated D25 neural rosettes (a) and D50 neurons (b–h) via dSMADi method with 12 days of patterning cues of Noggin (500 ng/ml) and SB431542 (10 μM) in addition to FGF2 (20 ng/ml, D12-D16) (Shi et al., 2012a). (C) Representative immunofluorescence images for induced neurons on D50 cocultured with mouse glia by NGN2 method (Marco de la Cruz et al., 2024; Zhang et al., 2013). Scale bars: 200 μm, 20 μm. Markers are mentioned in the main text.

Pluripotent stem cells possess the capacity to differentiate into derivatives of all three germ layers, including the neuroectoderm, by applying developmental principles and morphogen signaling cues identified through embryology (Das et al., 2020). A widely used strategy for neural induction is the dual SMAD-inhibition protocol, which suppresses BMP and TGF-β/Activin/Nodal pathways and is often combined with retinoids to promote dorsal forebrain specification (Chambers et al., 2009; Shi et al., 2012b). See Fig. 2A–B for example. Under these conditions, PSCs recapitulate cortical development through a well-defined series of steps: pluripotent cells (marked by OCT4, SOX2, and NANOG) first commit to cortical progenitor fates (marked by the expression of transcription factors such as PAX6, EMX1, and TBR2 and the intermediate filament NESTIN, Fig. 2B (a)) undergo extended phases of neurogenesis (immature neurons marked by expressing DCX and TUJ, Fig. 2B (c-h)) and subsequently subtype-specific cortical projection neurons characterized by markers such as TBR1, CTIP2, BRN2, and SATB2 as well as the dendrite marker MAP2 and neuron nuclear marker NEUN (Fig. 2B (b-h)). With further maturation, neurons develop synaptic and activity-dependent proteins, including PSD95, SYN1, and VGLUT1 (Fig. 2B (b-c)), and finally, glial differentiation produces astrocytes expressing GFAP and S100β after around 100 days *in vitro*. On the transcriptome level, cortical neurons derived from hiPSCs with the dual SMAD-inhibition protocol follow a similar developmental trajectory as seen *in vivo* (Sundaresh et al., 2025), which correspond to post-conception week 8–9 (56–63 days) of primary motor-sensory cortex (Mehta et al., 2018).

Electrophysiological and calcium (Ca^2+^) imaging studies demonstrate that hPSC-derived cortical neurons exhibit progressive functional maturation during extended *in vitro* culture (Kirwan et al., 2015). Over the first two months of differentiation, neurons show gradual hyperpolarization of the resting membrane potential, increased action-potential amplitude, and higher firing frequency, indicating enhanced excitability. Around day 40, neurons display immature dendritic spines, appearing as thin filopodial protrusions (∼2–5 μm) and begin to express functional NMDA receptors (Gupta et al., 2013). Ca^2+^ imaging confirms the emergence of activity-dependent Ca^2+^ transients, which are abolished by tetrodotoxin, verifying their dependence on voltage-gated sodium channels and neuronal firing.

At the network level, hPSC-derived cortical cultures follow a stereotyped developmental trajectory: initially uncoordinated spiking evolves into synchronized oscillatory bursting at approximately 8–10 weeks *in vitro*, followed by a gradual decline in synchrony and the emergence of complex, desynchronized firing patterns after around three months. By day 110, neurons display morphologically mature dendritic spines with thin necks (∼1–6 μm) and round heads (Kirwan et al., 2015). This temporal progression parallels key stages of human cortical network maturation *in vivo*, underscoring the value of hPSC-derived neural systems as tractable models for investigating early human neurodevelopment and circuit formation.

The original dual SMAD-inhibition protocol has since been modified in several ways to refine regional patterning and enhance neuronal maturation. For example, inhibition of Wnt signaling can promote anterior patterning (Rosebrock et al., 2022), while stimulation of sonic hedgehog (SHH) signaling drives ventral forebrain identities (Maroof et al., 2013). In addition, coculturing neurons with supporting glia accelerates functional maturation (Hedegaard et al., 2020). It is worth noting that primary rodent glia has been widely used as supporting cells in co-culture systems with human induced neurons, which can complicate interpretation due to species-specific differences (Degl'Innocenti and Dell'Anno, 2023). Conversely, human stem cell–derived glial cells commonly display prolonged maturation timelines *in vitro*, which can limit their ability to promote robust neuronal maturation and often results in less pronounced pro-maturational effects compared with rodent glia (Hedegaard et al., 2020; Rhee et al., 2019). Using such optimized approaches, Burke et al. differentiated hPSCs from multiple healthy donors for up to eight weeks and compared the resulting cultures to bulk RNA-sequencing data from human brain tissue (Burke et al., 2020). In agreement with earlier microarray studies, most cellular identities in these cultures corresponded to prenatal brain tissue, although a subset of cells displayed transcriptional and electrophysiological signatures of later developmental stages, indicating partial progression toward postnatal-like maturity (Stein et al., 2014).

An alternative strategy for neuronal differentiation is the direct induction of cortical neurons by exogenous expression of neuronal transcription factor(s), for example, *NEUROG2* (*NGN2*) (Zhang et al., 2013). See Fig. 2A–C for example. Single-cell transcriptomic benchmarking of NGN2-induced neurons at 15 and 21 days post-induction revealed strongest similarity to early excitatory neurons of the fetal cortex rather than to mature adult neurons (Das et al., 2024). Although these induced neurons form functional synapses, they generally lack dendritic spines and NMDA-receptor expression. Using a five-transcription-factor approach, Lin et al. reported the appearance of spine-like structures after approximately 2.5 months (Lin et al., 2023). Because NGN2 induction bypasses early progenitor stages including neuroectoderm and neuroepithelium induction (Lin et al., 2021), such models are well suited for studying postmitotic neuronal phenotypes, but less appropriate for investigating developmental or progenitor-related mechanisms (Shan et al., 2024). Compared with conventional dual-SMAD inhibition protocols, which typically require ∼4–6 weeks to generate neurons with mature electrophysiological properties, exogenous transcription factor–based differentiation can accelerate neuronal identity and functional maturation to similar phenotypic states within ∼2–3 weeks in two-dimensional cultures. Adding inhibitors to SMAD proteins and Wnt to cultures of induced neurons potentially represents a fruitful way forward by adding regional identity and promoting maturation (Nehme et al., 2018). By purifying neurons expressing CAMK2A, the authors demonstrate mature cells with NMDAR-mediated synaptic transmission.

### 5.2 Three-dimensional organoids

To generate a more physiological environment for cells during development, three-dimensional culture systems have been proposed. Lancaster et al. published the first protocol generating cortical brain organoids from pluripotent stem cells (Lancaster et al., 2013). This original paper has been followed by multiple studies on so-called unguided organoids, where each culture contains cells of different origin, as well as by the development of improved culture techniques (Sutcliffe et al., 2024). Importantly, cell differentiation in these systems is reproducible, leading to similar final cell-type compositions across organoids (Velasco et al., 2019). In parallel, more directed protocols have been developed that generate cortical organoids mimicking the dorsal forebrain, as well as organoids corresponding to other brain regions (Yoon et al., 2019).

Transcriptomic comparisons between human iPSC-derived neurons in organoids and primary brain tissue consistently show that most differentiation protocols yield cells resembling second-trimester fetal cortex (see Fig. 1C). An early study using single-cell RNA sequencing compared human cerebral organoids with fetal neocortex and found that organoids contained multiple progenitor and differentiated cell types from neuronal and mesenchymal lineages, with subsets corresponding to fetal neocortical regions (Camp et al., 2015). These cortical cells displayed gene-expression profiles closely matching those of fetal brain tissue, supporting their organization into cortex-like structures. After prolonged culture for up to nine months, organoids contain more mature neurons exhibiting dendritic spines and spontaneous network activity (Quadrato et al., 2017; Velasco et al., 2019). More recent work has shown that extended culture of human brain organoids can promote maturation toward early postnatal developmental states, as evidenced by multi-omic and functional transitions, although adult-like maturation remains incomplete (Gordon et al., 2021).

More recently, a consortium compared single-cell transcriptomic profiles of organoids generated using 26 protocols across 36 datasets (He et al., 2024). Early organoids, within the first three months of differentiation, most closely mirrored first-trimester human brain tissue, whereas prolonged culture produced profiles resembling second-trimester cell states. However, markers of late-stage neuronal diversification and maturation were largely absent, suggesting that current neural organoid approaches remain limited in their capacity to model postnatal or adult neuronal development.

Beyond transcriptomic maturation, three-dimensional brain organoids exhibit progressive functional maturation at the electrophysiological and network levels. Multiple studies have shown that cortical organoids develop spontaneous neuronal firing, synaptic activity, calcium transients, and episodes of synchronized network bursting that resemble early developmental patterns observed in the fetal and neonatal human brain (Gordon et al., 2021; Quadrato et al., 2017; Trujillo et al., 2019). These emergent network dynamics generally increase with prolonged culture and reflect self-organized circuit assembly. Compared with two-dimensional neuronal cultures, organoids better support population-level synchronization and recurrent network activity, albeit with greater heterogeneity.

## 6 Mechanisms constraining or promoting maturation

### 6.1 Inherent metabolic brakes

The fetal bias of iPSC based neural models reflects both the reprogramming reset – erasure of age-related epigenetic marks – and features of *in vitro* differentiation, which lacks cues from glia, vasculature, and long-range activity patterns. Notably, induced neurons from human pluripotent stem cells show slower maturation than equivalent ape models from bonobos and chimpanzees, paralleling the evolutionary delay in human neuronal differentiation (Schornig et al., 2021). Even when transplanting human PSC derived cortical neurons into mouse brain, neurons show prolonged maturation (Espuny-Camacho et al., 2013; Linaro et al., 2019), providing direct evidence for cell-intrinsic timing.

Human developmental processes progress at a markedly slower pace than those of other species. Differentiation of human stem-cell-derived neurons occurs approximately two-to three-fold more slowly than in the mouse, attributable to increased protein stability and prolonged cell-cycle duration (Rayon et al., 2020). Complementarily, Díaz-Cuadros et al. identified reduced metabolic activity and protein-synthesis rates as key determinants of this slower developmental tempo (Diaz-Cuadros et al., 2023). In line with this, neuronal maturation depends on the mitochondrial metabolic activity (Iwata et al., 2023). Comparative studies of neuronal differentiation from PSCs indicate that human developing cortical neurons exhibit lower mitochondria-driven tricarboxylic acid (TCA) cycle activity and reduced oxidative metabolism compared with mouse neurons at equivalent developmental stages. During differentiation, both human and mouse pluripotent stem cells transition from glycolytic to oxidative metabolism, but the timing and magnitude of this shift diverge between species. Mitochondrial activity was further enhanced by enriching the neuronal culture medium with free fatty acids, which, upon β-oxidation, supply additional substrates to the TCA cycle, thereby sustaining oxidative metabolism even under conditions of glycolysis (Iwata et al., 2023). This treatment led to accelerated neuronal development, with human neurons reaching maturation milestones several weeks earlier than controls. These neurons exhibited enhanced dendritic growth and arbor complexity, together with a higher density of synaptic connections. Functionally, they displayed earlier acquisition of electrical excitability and synaptic transmission, reflecting a global acceleration of neuronal maturation driven by elevated mitochondrial metabolism.

Together, these findings establish a mechanistic link between cellular metabolism, protein turnover, and species-specific developmental timing, providing an explanation for the protracted maturation and fetal-like state typically observed in human stem-cell-derived neural models. Understanding these constraints is crucial for interpreting disease-relevant phenotypes observed in iPSC systems.

### 6.2 Inherent epigenetic brakes

Several molecular and epigenetic “brakes” limit neuronal aging *in vitro.* Recent CRISPR screens identified Menin and SUZ12 as regulators of developmental timing, with knockout accelerating neuronal maturation (Xu et al., 2025). A complementary study implicated neddylation pathways in maintaining youthful neuronal states (Saurat et al., 2024). At the chromatin level, an epigenetic barrier involving the Polycomb complex was shown to set maturation timing in human neurons (Ciceri et al., 2024; Ciceri and Studer, 2024). Beyond genetic manipulations, transcriptomic aging clocks have been proposed to estimate and modulate “cellular age” in culture (Zhang et al., 2025). Together, these approaches outline a toolbox for systematically shifting iPSC-derived neurons along the developmental timeline.

**Fig. 3:**
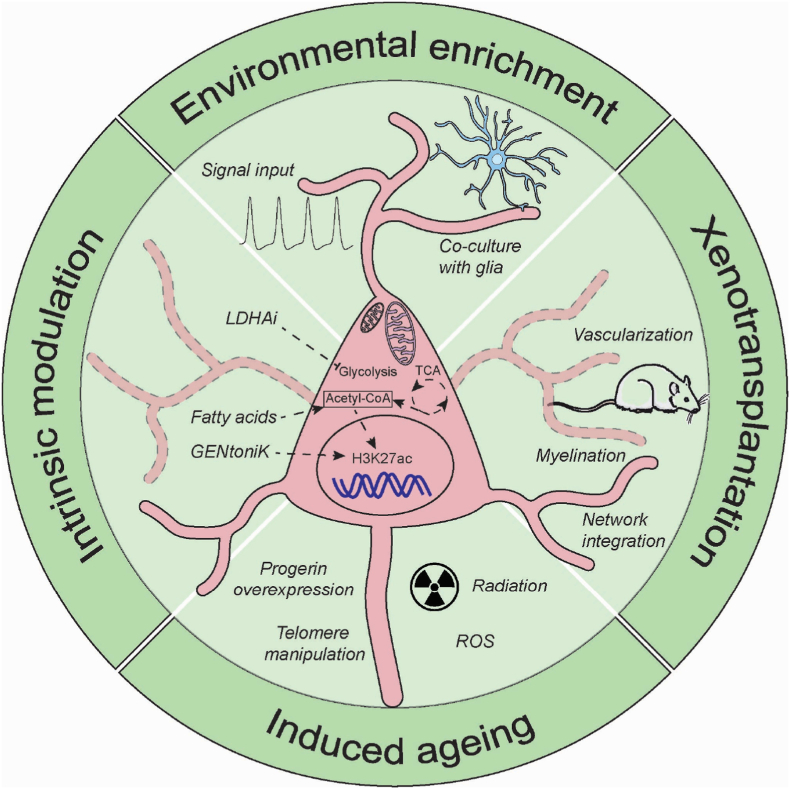
Cartoon over human neuronal maturation. Over time, mitochondria grow and shift from using mainly glycolysis to oxidative phosphorylation via the TCA cycle. In parallel, cells become hyperpolarized and exhibit spontaneous action potential firing. Maturation can be reached via four pathways: 1) enrichment of cell culture conditions with media supplements and electrical stimulation, 2) supplementation of fatty acids to promote epigenetic changes, 3) transplantation of *in vitro* cells to rodent brain, and 4) accelerated aging using expression of progerin.

Epigenetic and metabolic processes are tightly interconnected: while chromatin modifiers control developmental timing, cellular metabolism and protein turnover can, in turn, regulate epigenetic states (Ciceri and Studer, 2024). Several TCA cycle metabolites act as cofactors for histone-modifying enzymes (Schvartzman et al., 2018). Acetyl-CoA provides substrate for histone acetylation (e.g., H3K27ac), which increases during neuronal maturation (see Fig. 3). α-Ketoglutarate (α-KG) supports histone demethylases such as KDM5 and KDM6, linking mitochondrial metabolism to chromatin remodeling. Manipulating these pathways by for instance, inhibiting fatty acid oxidation or supplementing fatty acids, alters α-KG availability and thereby affects histone methylation and neuronal maturation (Iwata et al., 2023). Other TCA intermediates, including succinate and fumarate, can inhibit demethylases, further coupling metabolic flux to chromatin state (Schvartzman et al., 2018).

Studer's group also demonstrated that a defined small-molecule cocktail potentially can accelerate maturation and promote adult-like gene expression (Hergenreder et al., 2024). The epigenetic probes GSK2879552 and EPZ-5676 promote a transition in chromatin accessibility from an immature, migration- and axon guidance-associated profile to a more mature transcriptional program characterized by synaptic transmission and ion channel expression. They further proposed that this remodeled chromatin landscape enhances the capacity for NMDA- and Bay K 8644-evoked Ca^2+^-dependent transcription, thereby providing an additional mechanism driving neuronal maturation.

### 6.3 Xenotransplantation

Yet another method to putatively improve maturation is by transplanting human neurons into the brain of another animal. This way, human cells will presumably be provided with both neuronal activity and support from blood vessels and diffusing factors. For instance, cortical organoids grafted into rodent brains acquire vascularization, myelination, and mature synaptic responses over several months (Revah et al., 2022). One earlier study demonstrated how transplantation into the cerebral cortex of mouse brains reduces gene expression associated with stress and promotes regional differentiation of radial glia cells (Bhaduri et al., 2020).

### 6.4 Artificial aging

A complementary approach to enhance neuronal maturity in hPSC models is the introduction of age-associated factors, most notably progerin, a truncated form of lamin A that causes Hutchinson–Gilford progeria syndrome (diseases that cause individuals to age faster than usual) (Jothi and Kulka, 2024). During reprogramming, iPSCs erase most age-related epigenetic marks, reverting to a developmentally “young” state. Transient or moderate expression of progerin in iPSC-derived neurons partially restores age-related nuclear and chromatin features, including nuclear envelope irregularities, loss of heterochromatin, and accumulation of DNA damage, thereby producing a more adult-like cellular phenotype (Miller et al., 2013). Although excessive expression can be deleterious, controlled progerin induction offers a practical strategy to re-age neurons *in vitro* and bridge the developmental gap between fetal-like stem-cell derivatives and mature human brain tissue.

A potentially more physiological way of inducing aging is by manipulating telomere shortening, a core hallmark of aging (Vera et al., 2016). Telomerase is downregulated and telomeres shorten during neuronal differentiation, and this process can be accelerated pharmacologically in hPSCs and their derivatives. Neurons generated under these conditions display age-like features, including increased DNA damage, higher mitochondrial ROS and reduced dendritic complexity.

An important open question is how artificial aging strategies intersect with disease-relevant cell-type specification. While approaches such as progerin expression or telomere manipulation can induce age-associated cellular features, it remains unclear whether these neurons faithfully recapitulate the developmental and functional states corresponding to critical periods of vulnerability in neuropsychiatric disorders.

### 6.5 Rethinking immaturity as an opportunity

While limited maturity is often viewed as a drawback of iPSC-based models, it can be leveraged to probe early pathogenic mechanisms. Disorders with strong prenatal or perinatal origins, such as autism, intellectual disability, and certain epilepsies, are well suited to these systems. Moreover, even for adult-onset illnesses, fetal-stage cells can reveal primed vulnerabilities encoded in genetic or epigenetic programs that later interact with environmental stressors (Brennand et al., 2011). Conceptually, disease modeling might benefit from a developmental continuum, where iPSC systems represent the “first act” of pathology before clinical manifestation, establishing latent risk states that precede symptom emergence and may be obscured in later disease stages.

## 7 Conclusion

The developmental timing of mental disorders offers critical insight into their underlying biology, highlighting that pathogenesis unfolds within distinct temporal windows of human brain maturation. Patient-derived iPSC-based neural models have transformed psychiatric research by enabling direct investigation of human neurons carrying patient-specific genetic backgrounds. Yet, the intrinsic immaturity of most iPSC-derived neurons and organoids that correspond to mid-fetal stages poses interpretational challenges, particularly for disorders that manifest in adolescence or adulthood.

Importantly, large-scale integrative analyses of human brain development indicate that primary genetic risk mechanisms may be temporally and mechanistically distinct from transcriptional changes observed in diseased adult brains. PsychENCODE data demonstrate limited overlap between gene networks enriched for common genetic risk variants and those differentially expressed in *postmortem* tissue from individuals with schizophrenia, bipolar disorder, or autism spectrum disorder, suggesting that many disease-associated expression changes reflect secondary or compensatory processes rather than the initial effects of inherited risk. This dissociation underscores the importance of studying neurotypical brain development and cautions against equating late-stage molecular pathology with causal mechanisms.

Recent advances demonstrate that neuronal maturity is not a fixed limitation but a tunable property. Genetic and epigenetic regulators of developmental pacing have been identified through genome-wide CRISPR screens (Saurat et al., 2024; Xu et al., 2025), while targeted modulation of chromatin barriers can extend neuronal aging *in vitro* (Ciceri et al., 2024). Complementary physiological strategies, including prolonged culture, electrical stimulation, astrocyte co-culture, and *in vivo* transplantation, further enhance functional maturation (Diego-Santiago et al., 2025; Hergenreder et al., 2024; Revah et al., 2022). These emerging tools now make it possible to align model systems more precisely with the developmental epochs relevant to specific disorders. By contrast to two-dimensional, equivalent approaches for three-dimensional organoid systems to promote maturation remain less developed, reflecting their structural complexity and cellular heterogeneity, and represent an important area for future methodological innovation.

Moving forward, a nuanced view of cellular age is essential: fetal-like neurons may capture early developmental vulnerability, whereas more mature neuronal states may be required to model late-onset pathophysiology. Integrating developmental benchmarks – transcriptomic, electrophysiological, and epigenetic – with clinical metadata such as age of onset will provide a temporal framework for interpreting disease mechanisms. Ultimately, bridging developmental neuroscience with stem-cell biology promises not only to refine our models of psychiatric illness but also to reveal how the passage of developmental time shapes the architecture of mental health and disease.

## Declaration of generative AI and AI-assisted technologies in the manuscript preparation process

During the preparation of this work the authors used ChatGPT in order to format the text. After using this tool, the authors reviewed and edited the content as needed and take full responsibility for the content of the published article.

## Conflict of interest

The authors declare that they have no known competing financial interests or personal relationships that could have appeared to influence the work reported in this paper.
